# The Effect of Kosher Determinants of Beef on Its Color, Texture Profile and Sensory Evaluation

**DOI:** 10.3390/ijerph20021378

**Published:** 2023-01-12

**Authors:** Jagoda Żurek, Mariusz Rudy, Renata Stanisławczyk, Paulina Duma-Kocan

**Affiliations:** 1 Department of Financial Markets and Public Finance, Institute of Economics and Finance, College of Social Sciences, University of Rzeszow, Cwiklinskiej 2, 35-601 Rzeszów, Poland; jzurek@ur.edu.pl; 2 Department of Agricultural Processing and Commodity Science, Institute of Food and Nutrition Technology, College of Natural Sciences, University of Rzeszow, Zelwerowicza 4, 35-601 Rzeszów, Poland; rstanislawczyk@ur.edu.pl (R.S.); pduma@ur.edu.pl (P.D.-K.)

**Keywords:** ritual slaughter, kosher beef quality, color changes, sensory properties, texture profile

## Abstract

Traditional ritual slaughter is an underlying practice in religious Jewish communities and it translates into eating in accordance with core beliefs. This paper aims to comprehensively analyze factors determining the kosher status of beef (sex, slaughter method, and muscle type) and assess their impact on selected quality properties, such as color or texture profile. Sensory evaluation was also performed. The muscles were obtained from 80 carcasses (50% of heifers and 50% of young bulls). The experimental results indicate that each studied kosherness determinant influenced beef quality properties. The process of koshering caused the darkening of beef and lowered the share of color parameters red (a*) and yellow (b*). The influence of the type of slaughter on the values of adhesiveness, gumminess, and chewiness of beef was confirmed; higher values were mostly obtained in the muscles of cattle from kosher slaughter. As to sensory evaluation, the study showed that in the case of muscle type, the sex and slaughter method positively influenced only some of the properties.

## 1. Introduction

Slaughtering is a complex process that can induce stress in animals. Hence, stunning is often used in slaughterhouses to make animals unconscious while cutting their neck and bleeding without causing distress or pain. However, in many countries, slaughtering animals without stunning is widely practiced due to religious requirements [1].

Kosher slaughter of animals and meat processing is carried out in accordance with rabbinic law, in which stunning before slaughter is not acceptable [2]. The animal must be conscious at the time of slaughter. According to Jewish dietary laws [3], koshering is the final step in the process of making meat fit for consumption by Jewish consumers. In koshering, blood is removed from the meat, and then the meat is soaked in water for half an hour, salted with coarse salt for an hour, and rinsed thrice [4].

The quality of beef depends on various factors. Some of the most important ones are breed, growth rate, nutrition, gender, age, and animal handling before and during slaughter [5,6,7,8,9]. The intramuscular fat (IMF) content affects meat tenderness and juiciness [10,11,12], whereas the muscle fiber composition affects its color, tenderness, and final pH [13,14]. Minimal amounts of IMF are necessary for the flavor and tenderness of beef [15].

The color of meat is important to consumers at the time of purchase as it is closely associated with its freshness. Consumers consider beef color as one of the most important parameters while purchasing beef. However, meat quality and color are not well correlated [16,17]. Meat color is determined by the final pH value after the slaughter and the level of myoglobin in the muscle [18]. Storing meat at reduced temperatures has a beneficial effect on its color by increasing its oxygen solubility [19,20,21]. Dark meat is more susceptible to bacterial spoilage and has a lower shelf life and reduced flavor compared with normal meat [22]. One of the major issues in koshering is the loss of the red color of meat. Because high salt concentrations promote the oxidation of myoglobin molecules, meat subjected to salting usually has an unattractive brownish-gray color [23].

Meat parameters that affect its quality, such as color, flavor, tenderness, juiciness, and traits related to water-holding capacity, affect consumer satisfaction [15,24]. The problem of reduced meat quality caused by preslaughter handling is observed more frequently in bull meat than in heifers and steers [5]. Lowe et al. [25] indicated a correlation between stress response in bulls and meat quality, which is primarily due to the low glycogen content. Bleeding of animals during slaughter should be abundant and complete and should occur in the shortest possible time. Blood lingering in the tissues is a buffering factor that counteracts postslaughter acidification and reduces the bacteriological stability of meat, thus affecting the development of desirable sensory characteristics [26]. In addition, cooling carcasses after slaughter is an important component of the meat procurement process that affects the hygienic, technological, and sensory properties of meat [27].

Breeding bulls is a challenging task due to their aggressive and sexual behavior. Bulls are more susceptible to preslaughter stress, which can result in meat with high pH and poorer quality traits such as dark muscle coloration and less tender cutouts [28,29].

Few studies in the literature have reported the influence of factors related only to kosher slaughter on the quality traits of beef. Taking this fact into account along with the aforementioned observations, this study was carried out to comprehensively analyze factors such as slaughter type, gender, and muscle type, which are decisive in the koshering of beef (kosher determinants), and their influence on the color, texture, and sensory characteristics of this raw material was determined. This study will bridge the gaps in our knowledge regarding meat quality and significantly expand our understanding of the influence of factors related to the specifics of kosher slaughter on selected beef quality characteristics. Since meat is a highly variable product determined by numerous genetic and environmental factors, previous studies conducted under different conditions have not solved the problems now posed. A new study may reveal new trends or confirm the existing results.

## 2. Materials and Methods

### 2.1. Raw Material

The study sample consisted of two types of muscles: the longissimus thoracic muscle (musculus longissimus thoracis, MLT) and the supraspinatus muscle (musculus supraspinatus, MS). They were obtained from carcasses of heifers and young bulls (20 carcasses each). The average live weight of 20 heifers and 20 young bulls selected for traditional slaughter was 520 ± 58 and 591 ± 52 kg, respectively, and the average live weight of 20 heifers and 20 young bulls selected for kosher slaughter was 531 ± 60 and 571 ± 59 kg, respectively. The specifications for animals and the method of obtaining the raw material were the same as presented in our previous study [30]. According to kosher rules, Jews eat meat obtained from forequarters. Therefore, muscle samples from this batch of carcasses were taken for the research. In addition, the muscles that performed more physical activity during the life of the animal as compared to less active ones can be differentiated in terms of color parameters. Therefore, the supraspinatus muscle was chosen for this study, as it performs more work than the longissimus thoracis.

### 2.2. Analytical Methods

This study was conducted in the half-carcasses of beef, from both traditional and ritual slaughter. Analyses and measurements were carried out in temperature-controlled rooms while maintaining a temperature regime of 0–2 °C. Approximately 0.5 kg of the samples was taken from the muscles 48 h after slaughter for laboratory testing, which included the determination of texture parameters, measurement of color parameters, and sensory evaluation of the meat. Muscle color parameters were measured again 72 h after slaughter.

Texture profile analysis was carried out using a Texture Analyser CT3-25 from Brookfield with a 38.1 mm diameter, 20 mm long cylindrical attachment. This analysis consisted of a double compression test of cube-shaped samples with side lengths of 20 mm, up to 50% of their original height. The cylinder displacement speed was 2 mm/s, and the interval between two compressions was 2 s. The following texture parameters were determined: hardness (hardness 1—the maximum value of the force measured during the first compression cycle of the sample; hardness 2—the maximum value of the force measured during the second compression cycle of the sample), rigidity (rigidity 1—the force required to deform the sample for a distance of 5 mm; rigidity 2—the force required to deform the sample for a distance of 8 mm), adhesiveness, resilience, gumminess, chewiness, cohesiveness, and springiness. The results of texture parameter measurements were determined automatically using the Texture Pro CT software.

Instrumental measurements of color parameters in the CIE L*a*b* system were carried out using a HunterLab UltraScan PRO electronic spectrophotocolorimeter (light source D65, measuring head aperture 8 mm, calibration with white standard: L* 99.18, a* 0.07, and b* 0.05). In this system, L* represents brightness, which is a spatial vector, whereas a* and b* are trichromaticity coordinates, where positive values of a* correspond to red and negative values to green, and positive values of b* correspond to yellow and negative values to blue. The difference in the individual color parameters (ΔL; Δa; Δb) after 24 h of refrigerated storage was also calculated, starting 48 h after slaughter, and the absolute color difference (ΔE), which is the Euclidean distance between two points in three-dimensional space, was calculated using the following formula:

$$\Delta E=\sqrt{{\left(\Delta L\right)}^{2}+{\left(\Delta a\right)}^{2}+{\left(\Delta b\right)}^{2}}$$

It can be assumed that the observer notices the color difference as follows:

- 0 < ΔE < 1—does not notice the difference;
- 1 < ΔE < 2—only an experienced observer notices the difference;
- 2 < ΔE < 3.5—an inexperienced observer also notices the difference;
- 3.5 < ΔE < 5—notices a distinct color difference;
- 5 < ΔE—an observer gets the impression of two different colors.

The sensory qualities of MLT and MS obtained from cattle after standard and kosher slaughter were determined using the method of Baryłko-Pikielna and Matuszewska [31]. Of each muscle sample, 100 g was steamed at 95 °C until an internal temperature of 80 °C ± 2 °C was achieved. The temperature was measured using a digital thermometer with a needle probe (Sous Vide Thermapen, MERA, Warsaw, Poland). For the sensory assessment, the heat-treated samples were cooled up to 20 °C ± 2 °C and cut into 1.5 cm thick slices, perpendicular to the run of meat fibers. They were placed in disposable plastic boxes covered with lids. Then, all samples were coded individually in a random order. Panelists evaluated each sample in triplicate. The sensory evaluation was carried out by a 6-person evaluation team (50% female/male in the 26–46 age range), in which sensory and sensitivity fitness was tested according to ISO, 8586-2:2008 [32] and ISO, 8587:2006 [33]. The panelists had experience in assessing meat products and meat. A 5-point evaluation was used with a defined value limit, including the following qualitative indices:

- aroma intensity (1 = very negative, very poorly perceptible; 5 = very strong),
- taste intensity (1 = very negative, very poorly perceptible; 5 = very desirable),
- aroma desirability (1 = not desirable, 5 = highly desirable),
- taste desirability (1 = not desirable, 5 = highly desirable),
- juiciness (1 = very dry, 5 = very juicy),
- tenderness (1 = very hard, 5 = very tender).

The color was measured 48 and 72 h after slaughter to check whether the koshering process had any influence on the changes in the tested parameters during 24 h cold storage.

All the evaluations were performed in a sensory laboratory in accordance with the relevant standard requirements [34]. Between each evaluation, the panelists took a break, for 30 s. During the break, they rinsed their mouths using mineral water. A total of 10 sessions were conducted with 16 samples per session.

### 2.3. Statistical Analysis

All experiments were performed in triplicate. The results obtained were assessed using statistical methods. Data were analyzed using a three-way analysis of variance (ANOVA) to determine the differences in the physical and chemical properties of beef, which were found to be affected by slaughter type, gender, and muscle type. To determine the effects of these parameters on the quality of the final product, the general linear model procedure was used (ANOVA, STATIST ICA version 13.1; StatSoft, Krakow, Poland) for a fixed effect model with two types of slaughter, two groups of muscle, and two groups of gender. In the case of significant effects (*p* < 0.05), the average values were compared using Tukey’s post hoc HSD test (ANOVA, STATISTICA version 13.1; StatSoft, Krakow, Poland). The average values and the standard error of mean values of color parameters, texture parameters, and sensory properties of beef samples are summarized in Table 1, Table 2 and Table 3.

## 3. Results

Slaughter type had a statistically significant effect on all meat color parameters analyzed after 48 and 72 h of refrigerated storage (Table 1). Muscle type had a major effect on the proportion of red color a*_48_ and a*_72_. The interaction effect between gender and slaughter type affected the brightness of meat color L*_72_. Furthermore, the interaction effect between muscle type and slaughter type had a statistically significant effect on meat color parameters a*_72_ and b*_72_. In both MLT and MS, higher values of L*_48_ color brightness (*p* < 0.05) were observed in cattle subjected to standard slaughter compared with those obtained from the carcasses of animals subjected to kosher slaughter. Higher values of the meat color parameter a*_48_ (*p* < 0.05) were observed in MS and MLT of conventionally slaughtered cattle compared with those obtained from the carcasses of animals subjected to kosher slaughter. In contrast, in cattle subjected to standard slaughter, higher values (*p* < 0.05) of a*_48_ were observed for MS than for MLT. Statistically significantly higher values of the parameter b*_48_ were observed in MS of cattle subjected to conventional slaughter compared with those from the carcasses of bulls subjected to ritual slaughter. Higher values of color brightness L*_72_ (*p* < 0.05) were observed in MLT of bulls subjected to standard slaughter than in those from the carcasses of ritually slaughtered cattle. However, in MS, higher L*_72_ color brightness values (*p* < 0.05) were observed in the carcasses of conventionally slaughtered cattle compared with heifers and bulls subjected to kosher slaughter. In both MLT and MS, higher values of the color parameter a*_72_ (*p* < 0.05) were observed in the muscles of cattle subjected to standard slaughter compared with those obtained from the carcasses of ritually slaughtered animals. Statistically significantly higher values of the meat color parameter a*_72_ were observed in MS of heifers and bulls subjected to standard slaughter compared with MLT obtained from the carcasses of animals of the same gender following the same type of slaughter. Statistically significant differences in the meat color parameter b*_72_ were observed between cattle subjected to standard slaughter and animals subjected to kosher slaughter, and between bulls and heifers following kosher slaughter.

For all beef muscle color parameters tested, lower values of brightness L* and proportions of red a* and yellow b* color were observed for meat from heifers and bulls subjected to kosher slaughter, compared with those determined in the raw material obtained from carcasses of cattle subjected to standard slaughter.

Slaughter type and the interaction effect between gender and muscle type had a statistically significant effect on meat texture parameters such as adhesiveness, gumminess, and chewiness (Table 2). In addition, the interaction effect between gender and muscle type affected the variation in rigidity 1 and 2. Muscle type and the interaction effect between gender and slaughter type also had a statistically significant effect on adhesiveness. The interaction effect between gender, muscle type, and slaughter type affected hardness 1 and 2 and rigidity 2 of beef.

While analyzing meat texture parameters, higher (*p* < 0.05) values were observed for the hardness of MLT of bulls subjected to ritual slaughter compared with those obtained from the carcasses of animals of the same gender subjected to standard slaughter. In MS, statistically significantly higher hardness values were determined in the raw material from the carcasses of heifers after kosher slaughter compared with those obtained from the carcasses of bulls after standard slaughter. In addition, in standard slaughter, higher (*p* < 0.05) values were observed for hardness 1 and rigidity 2 in MS of animals than in MLT of heifers and bulls from the same type of slaughter. MS of heifers subjected to standard slaughter showed statistically significantly higher values of rigidity 1 than that of bulls subjected to ritual slaughter. In addition, in standard slaughter, higher values of this parameter (*p* < 0.05) were observed in MS of heifers than in MLT of animals of the same gender. Higher values of rigidity 2 (*p* < 0.05) in MS were observed for heifers subjected to kosher slaughter than in bulls from both types of slaughter. Furthermore, higher adhesiveness values were observed in MS of bulls subjected to kosher slaughter than in animals of the same gender subjected to standard slaughter. However, statistically significantly higher values of adhesiveness and lower gumminess and chewiness were observed in MLT of heifers subjected to conventional and ritual slaughter than in MS of animals of the same gender after both types of slaughter. Higher values of gumminess and chewiness were observed in both MS and MLT of animals subjected to kosher slaughter than in those of heifers and bulls subjected to standard slaughter, but statistically significant differences were determined only between selected groups. The typically higher values of hardness, rigidity, gumminess, and chewiness (albeit not always statistically significant) of cattle from kosher slaughter as compared with the raw material obtained from carcasses of animals from standard slaughter, resulted from the impact of salt, which by acting on meat proteins could probably cause their partial coagulation (salting out). Moreover, the higher values of hardness 1 and rigidity in MS muscle versus MLT muscle of cattle from standard slaughter likely stemmed from the higher amount of collagen determined in the muscle exercising greater physical activity during the life of an animal.

Gender had a statistically significant effect on flavor intensity and tenderness of beef (Table 3). Slaughter type, muscle type, and the interaction effect between slaughter type and gender, and between gender and muscle type affected (*p* < 0.05) the tenderness and juiciness of the samples. In addition, the type of slaughter had a statistically significant effect on the intensity of flavor and its desirability.

In both standard and kosher slaughters of cattle, a statistically significantly higher flavor intensity was observed in the muscles of bulls compared with heifers. The desirability of this trait was also higher for meat from bulls than that from heifers, but statistically significant differences were not observed in this case. Sensory evaluation showed that MLT was characterized by a higher tenderness (*p* < 0.05) than MS, whereas both muscles obtained from heifer carcasses showed a higher tenderness (*p* < 0.05) than those from bulls, regardless of the type of slaughter. In our previous study [30] on the raw materials in question, higher cutting force values (*p* < 0.05) were observed in MS of cattle compared with MLT. In addition, statistically significantly higher values of this trait were observed in both muscles for bulls compared with heifers, regardless of slaughter type. MS and MLT of cattle subjected to conventional slaughter were characterized by higher cutting force values (*p* < 0.05) than those of carcasses of ritually slaughtered animals.

## 4. Discussion

After 24 h of refrigerated storage, the values of color brightness L*_72_ of MLT increased for meat from bulls and heifers subjected to kosher slaughter, whereas these values decreased slightly for the raw material obtained from the carcasses of bulls subjected to standard slaughter. On the other hand, for MS, the values of color brightness L*_72_ decreased after 24 h of refrigerated storage in meat from cattle subjected to standard slaughter and bulls subjected to ritual slaughter, whereas for the raw material obtained from the carcasses of heifers subjected to kosher slaughter, these values increased. The proportion of red color a* after 24 h of refrigerated storage of MLT and MS increased to a greater extent in bulls and heifers subjected to standard slaughter than in the raw material obtained from the carcasses of cattle subjected to kosher slaughter. A similar relationship was observed for the proportion of yellow color b* after 24 h of refrigerated storage for both muscles obtained from the carcasses of animals subjected to standard slaughter. In MS of heifers subjected to kosher slaughter, the proportion of yellow color b* decreased after 24 h of refrigerated storage.

The absolute color difference (ΔE) of MLT of bulls subjected to kosher slaughter, calculated from the L*, a*, and b* components, was noticeable to an inexperienced observer, whereas a clear color difference was observed in the raw material from the carcasses of the other groups of cattle subjected to standard slaughter. However, the absolute color difference (ΔE) of MS of cattle subjected to standard slaughter was clear, whereas in the meat of bulls subjected to ritual slaughter, this difference was noticeable to an inexperienced observer. Furthermore, the absolute color difference (ΔE) in the meat of heifers subjected to kosher slaughter could be perceived by an experienced observer. The process of koshering resulted in the darkening of beef color and reducing the contribution of red (a*) and yellow (b*) color after 48 of refrigeration storage as compared with the raw material obtained from animal carcasses from normal slaughter. This was likely caused by the fact that the salt added during the koshering treatment resulted in the destabilization of muscle proteins and thus accelerated denaturation of these compounds, which increased their susceptibility to discoloration. However, the lower mean values of absolute color difference (calculated in the period from 48 to 72 h from slaughter) in muscles under refrigeration storage for 24 h from kosher slaughter demonstrate that the differences in the color of such beef are less noticeable in this period.

The color of fresh beef is a crucial quality parameter that influences the consumer’s decision at retail [35]. If the color is not acceptable to the buyer, all other characteristics of meat assessed visually will lose their importance [36]. Color as a visual impression is induced by the presence of pigments, although it also depends on the structure and tissue composition of the meat [35]. Consumers usually associate the change in meat color from bright red (oxymyoglobin) to brown (metmyoglobin) with spoilage of the raw material. Increased discoloration of meat is attributable to the presence of ultraviolet light in display cases, low pH of meat, high temperatures, and the presence of salt [37,38]. The total amount of light reflected from and absorbed by the surface of the meat slice determines the brightness of the color L*. The value of this parameter is largely dependent on the physical properties of the muscle tissue that determine its structure [39].

Meat color is strongly dependent on the pigment protein myoglobin, which is responsible for transporting oxygen from the blood to the muscles and storing it. Due to differences in energy requirements and metabolism, the concentration of this protein varies between species and also between muscles [40].

The color stability of different beef muscles varies during postslaughter cold storage [41]. Of the beef carcass muscles, *m. longissimus dorsi* is the most stable in terms of color, *m. semimembranosus* is less stable, *m. gluteus medium* is not very stable, whereas *m. psoas major* is the least stable [42].

Muscles more resistant to fatigue, or endurance muscles (i.e., muscles close to the bone), need oxygen because they are rich in mitochondria and use oxidative metabolism as a source of energy production. Glycolytic muscles are typically involved in rapid energy flow, and since they do not need oxygen to function, myoglobin abundance is reduced, thus giving them a paler or lighter color. In contrast, when myoglobin is present in abundance due to the muscle’s demand for oxygen, a deeper red color is observed [43,44].

Myoglobin levels in the skeletal muscles of cattle are influenced by their age and breed type, as well as their physical activity. The muscles of slaughtered cows contain higher myoglobin levels than those of heifers, bulls, or steers [45]. The older the cattle, the darker the color of the meat [46].

Insausti et al. [47] reported that meat with a higher pigment content is more susceptible to oxidation and its color is less stable. However, McKenna et al. [41] and Jeong et al. [48] showed that the stability of beef muscle color is determined by the type of muscle fibers, pH, the myoglobin content, and the concentration of endogenous anti- and pro-oxidants that affect the degree of myoglobin and lipid oxidation.

The process of meat koshering is associated with salting and is intended to remove blood from the meat [4]. During koshering, a certain amount of salt is dissolved and applied on the surface of the raw material, resulting in discoloration during storage [38].

Salt reduces the oxidative stability of meat products, thus leading to discoloration [49]. It can also accelerate the peroxidation of muscle lipids [37]. The addition of salt to meat, either before or after the postmortem rigidity, destabilizes muscle proteins and accelerates the denaturation of these compounds, which increases discoloration [50]. Salting has been found to have three effects on meat color [51]:

1. It acts as a pro-oxidant in the oxidation of heme pigment, causing browning of meat.
2. It has a denaturing effect.
3. It increases the water-binding capacity of meat proteins, making tissues darker.

Salting during meat koshering can reduce or inhibit the growth of microorganisms, including pathogens, due to the associated changes in water activity and ionic strength [52].

It has been reported that koshered meat has low color intensity [23,53]. The use of large amounts of salt results in low color stability and accelerates the discoloration of the meat, which can be mitigated using antioxidants such as sodium erythorbate, modified atmosphere packaging [53], and hydrodynamic pressure [23].

Holzer et al. [23] determined that in *longissimus lumborum* of steers subjected to koshering, the values of color brightness L* on the day of slaughter were 35.3, whereas the values of color parameters a* and b* were 15.8 and 6.0, respectively. In the present study, after 48 h of refrigerated storage of ritually slaughtered cattle muscles, values of color brightness L* similar to those reported by Holzer et al. [23] were observed, whereas the proportions of red color a* and yellow color b* were higher in the studied muscles after kosher slaughter. In the same study, darkening of the outer surface of koshered meat was observed on the 14th day of storage, whereas L* color brightness values increased in the control sample (without koshering).

In *longissimus thoracis* of bulls, Węglarz [54] reported that the L*_48_ color brightness values (39.74) were slightly lower than those obtained in the present study in the muscles of bulls subjected to standard slaughter, whereas the values determined in the muscles of heifers were slightly higher (41.41) than those obtained in the present study in the muscles of heifers subjected to standard slaughter. Similar results were reported by Rudy et al. [55] in *longissimus dorsi* of bulls and heifers. In addition, Węglarz [54] showed that the a*_48_ and b*_48_ color parameters (12.83 and 2.02) in MLT of bulls and heifers were lower than those obtained in the present study (14.10 and 3.25). In contrast, Rudy et al. [55] reported the values of these traits in beef muscle at the levels of 16.11 and 13.72, and 15.78 and 14.01, respectively, which indicates that the values of the a*_48_ parameters were lower and those of b*_48_ were higher than those obtained in the present study.

Onenc and Kaya [1] compared the effect of the type of stunning on the color of beef meat obtained from *longissimus thoracis et lumborum* of bulls 48 and 72 h after slaughter. They showed that both in the absence of stunning under the Turkish slaughter procedure and in the presence of electric stunning, the values of color brightness L* increased from 36.79 to 39.02 (slaughter without stunning) and from 36.78 to 37.27 (slaughter with electric stunning), respectively. In the present study (Table 1), similar results were obtained for the muscles of cattle subjected to kosher slaughter, except for MS of bulls, where the values of color brightness L* decreased after 24 h of refrigerated storage (from 48 to 72 h). These authors also showed that the color brightness of muscles obtained from animals stunned with a percussive captive bolt did not change with time (L = 41.00). Between 48 and 72 h after slaughter, they observed an increase in the proportion of red color a* in muscles obtained from ritual slaughter and with electric stunning (from 14.87 to 15.80; from 15.08 to 15.20, respectively), whereas they showed an increase in the proportion of yellow color b* only for meat obtained from animals slaughtered without stunning (an increase in values from 13.16 to 14.55). The proportion of yellow color b* of meat obtained from the carcasses of bulls stunned electrically and with a percussive captive bolt after 48 h was 13.32 and 15.70, respectively, whereas after another 24 h of refrigerated storage, these values were 12.99 and 14.83, respectively. Onenc and Kaya [1] reported statistically significant differences (*p* < 0.05) between groups only for the raw material 72 h after slaughter and only for L* and b* values. Vergara and Gallego [56] and Velarde et al. [57] showed no statistically significant differences in color parameters between electrically stunned (ES) and nonstunned lambs. In addition, Anil et al. [58] observed no statistically significant differences in color parameters of beef meat (*m. trapezius*) between groups slaughtered with captive bolt stunning and neck cutting without stunning (Halal). In contrast, in another study, statistically significant differences were observed in color parameters between different groups—meat from captive-bolt-stunned animals was the darkest, followed by meat obtained from lambs without stunning, whereas the lightest was from ES animals [59]. Zuckerman and Mannheim [53] showed a lighter color of *longissimus* before koshering compared with beef after the koshering process (L = 37.38, L = 32.92 (*p* < 0.05), respectively). They also observed a 24% increase in the proportion of red color before koshering, i.e., a = 16.94 vs. a = 12.88 after koshering (*p* < 0.01), and a 40% decrease in the proportion of yellow color after koshering (*p* < 0.01).

Vestergaard et al. [60] showed that differences in meat color may be attributable to, among others, the housing system of animals through changes in physical activity, which can affect muscle type and metabolism. In addition, nutritional factors or the breed of the animals can lead to differences in meat color [61,62]. Furthermore, higher rates of temperature drop and initial pH in the carcasses of animals subjected to standard slaughter can affect meat color [63].

Meat texture analysis is carried out by measuring the deformation due to the compression of the sample [64,65,66]. By definition, texture is a sensory parameter that can be perceived, described, and quantified by humans [67].

Meat texture is influenced by numerous live and postslaughter factors such as species, breed, and gender [21,47], as well as muscle type [68,69,70,71,72]. Meat texture determinants also depend on slaughter type and carcass cooling conditions, maturation parameters, and time, as well as on the creation of appropriate conditions for the course of postslaughter changes [73,74,75,76]. Stress during slaughter leads to a decrease in muscle glycogen levels, which in turn results in dark, tough, dry (DFD) meat. Heifers have a lower temperament than bulls and are less susceptible to stress. Their meat is less prone to the DFD defect [77]. Several studies have reported changes in the tenderness of raw beef [78,79]. However, an increase in tenderness has been observed 24 h after slaughter due to enzymatic degradation of muscle tissue caused by proteolytic enzymes, pH, the degree of crosslinking, and the amount of connective tissue, as well as animal species [78,79,80].

In cattle, gender significantly affects the texture of beef meat. This is attributable to the presence of IMF and the smaller diameter of muscle fibers in cows, making their meat more tender than that of bulls [81]. The biological structure of the skeletal muscle tissue is the most important parameter that determines the quality characteristics of meat (texture, tenderness, color, and water absorption) and its suitability for processing. [82,83]. The fat content of the muscle tissue is important for meat palatability and texture [5]. Factors such as sarcomere length and collagen contribute to the firmness of beef texture and may play an important role in the differences observed between the genders. In general, bulls have higher levels of collagen in their muscles and shorter sarcomeres [84].

Onenc and Kaya [1] reported statistically significantly higher values of hardness (13.83 kg) and gumminess (14.06 kg) 24 h after slaughter in *m. longissimus thoracis et lumborum* of ES cattle than in muscles obtained from animals without stunning (NS): 9.80 and 10.35 kg, respectively, and in muscles from those stunned using a percussive captive bolt (PS): 9.12 and 8.98 kg, respectively. In addition, they showed statistically significantly lower chewiness values in the muscles of PS cattle (9.77 kg/mm) than in the muscles of NS (15.52 kg/mm) and ES (14.18 kg/mm) animals 24 h after slaughter. They also showed that, with the time of maturation, a decrease in hardness values was observed in all the groups studied (NS—4.53 kg, ES—4.58 kg, PS—4.52 kg), which is in line with the studies of other authors [57]. Vergara and Galleo [56] showed that meat obtained from NS animals appeared more tender than that from ES animals, but the differences were not statistically significant.

Miciński et al. [85] reported lower hardness values in *longissimus* of Hereford and Limousine bulls 48 h after slaughter (31.98 and 49.61 N, respectively) than those obtained in the present study. In addition, they observed higher values of cohesiveness (0.553 and 0.552 N, respectively) and lower resilience (0.645 and 0.585 mm, respectively). They also reported lower values of gumminess (17.35 N) in the muscle of Hereford bulls; however, in the muscle of Limousine animals, they showed higher values of this trait (27.24 N) than in the muscle of cattle subjected to standard slaughter in the present study, except for MS of heifers, in which the values were higher (38.831 N).

Kosher slaughter showed a favorable effect on the tenderness of the beef meat tested. Sensory evaluation also showed better juiciness (*p* < 0.05) of meat obtained from animals subjected to ritual slaughter. A statistically significantly higher score for juiciness was observed in MLT of heifers than in MS of animals of the same gender, regardless of slaughter type. Furthermore, in standard slaughter, a statistically significantly higher juiciness score for both muscles was observed in heifers than in bulls. In our previous study [30], a lower water content and a higher fat content were observed in MLT of cattle than in MS of bulls and heifers, regardless of slaughter type. In addition, a higher fat content and a lower water content were observed in both MS and MLT of heifers (*p* < 0.05) than in those obtained from bull carcasses, regardless of slaughter type.

Higher scores (*p* < 0.05) were observed for intensity and desirability of the samples tested in cattle muscles after kosher slaughter compared with the raw material of those after standard slaughter (Table 3).

Many researchers have reported that beef intended for culinary purposes reaches its maximum organoleptic qualities and, above all, tenderness after a sufficiently long period of maturation—from at least 7–21 days at a temperature of about 2–4 °C [86,87,88]. Meat maturation naturally improves the flavor and tenderness of the whole carcass or its parts at refrigerated temperatures [89]. The final quality of the meat is also determined by how it is packaged [90,91]. Improper storage conditions and meat packaging techniques can lead to undesirable changes in the meat, reducing its quality indicators [92]. As well as maturation, the final palatability of beef is also determined by other factors such as both breeding and processing (e.g., breed, gender, animal age, muscle type, iron and myoglobin levels) [93,94,95,96,97,98].

Individual muscles vary in palatability. For example, *m. psoas*, the most tender muscle of cattle, is characterized by poor palatability. However, *m. psoas major* and *m. teres major* are characterized by more intense palatability than *m. rectus femoris*. A stronger flavor is observed for *m. longissimus dorsi* than for *m. semitendinosus*. The palatability of the diaphragm is particularly intense. Kukowski et al. [99] observed that *m. triceps brachii* is more flavorful than *m. longissimus thoracis* and that *m. serratus ventralis* and *m. complexus* are less aromatic than *m. supraspinatus*. Muscles with higher activity in the live period show a stronger aroma than muscles that are less active [29,100].

Meat tenderness is one of the most desirable parameters among beef consumers. As animals age, meat tenderness decreases, which is influenced by an increase in intramuscular connective tissue content [101].

The gender of cattle (female, male, castrated) affects the growth performance and carcass characteristics [102]. One of the key determinants of meat sensory properties is the IMF content, which characterizes the content of fat in skeletal muscle [103]. Castration contributes to an increase in the intramuscular fat content, thereby improving beef quality [104]. Marbling, or intramuscular and intermuscular fattening, imparts higher tenderness and juiciness to the meat due to higher loosening of the connective tissue due to an even distribution of fat in the muscle [105]. Steers and heifers produce finer meat with better sensory properties compared with meat obtained from bull carcasses, which is characterized by thicker muscle fibers [35,106]. Once males reach sexual maturity, testosterone, which is produced by testicular interstitial cells, has a positive effect on muscle development due to the activation of nitrogen retention. This results in a higher final weight, with better feed utilization [107]. The meat of bulls is characterized by a higher muscle-to-fat ratio and a higher yield by about 3–5%, compared with heifers and steers, but the carcasses of heifers and steers show a higher content of subcutaneous and intermuscular fat. Their fattening performance is inferior to that of bulls, but the carcasses of animals of this gender are qualitatively better due to the high IMF content that has a beneficial effect on the juiciness and flavor of the meat [77,108,109]. Furthermore, table salt (NaCl) affects the sensory properties of meat products [110].

Onenc and Kaya [1] showed that for all beef sensory traits—tenderness, flavor, aroma, and overall acceptability at all maturation periods, i.e., 24 h, 4, 7, and 14 days, percussive-stunned cattle showed statistically significantly higher scores than nonstunned animals.

Pogorzelska-Przybyłek et al. [111] showed a higher fat content in *longissimus thoracis* of steers and heifers compared with bulls, which positively influenced the sensory properties of beef meat. They found that in the sensory evaluation (in which 1 and 5 represent the minimum and maximum scores, respectively), heifer meat showed a statistically significantly higher score for tenderness (4.00) than bull meat (3.14), which is consistent with the present study (Table 3). Pogorzelska-Przybyłek et al. [111] showed statistically significantly higher scores for flavor and juiciness of the heifer muscle, 4.86 and 4.00, respectively, compared with bulls: 4.50 and 3.79. A higher score for tenderness in the case of muscle obtained from carcasses of heifers versus muscles obtained from the carcasses of young bulls probably originated from the fine fiber structure of muscles that is most frequently determined in muscles of individuals of the former sex. Moreover, the higher score for tenderness and juiciness of the meat of heifers could be caused by the higher intramuscular fat content, typically found in muscles of this sex as compared with young bulls.

Mueller et al. [112] investigated the effect of gender on quality traits of beef from the Brazilian Certified Angus Beef Program and reported that gender strongly affected all sensory traits (*p* < 0.05), which were tested after 14 days of maturation at 2 °C. Bull meat achieved the lowest overall consumer acceptability score (*p* = 0.0278). Beef obtained from carcasses of steers, heifers, and immunocastrates is considered to be of better quality than that obtained from bulls. Mueller et al. [112] and Gorraiz et al. [94] observed a stronger flavor in beef obtained from heifers than from bulls, which may be related to breed, carcass fat levels, sex hormone production, and their effect on lipid composition [113].

In the present study (Table 3), the higher overall acceptability of beef after kosher slaughter compared with standard slaughter may be attributable to the higher salt content of the raw material. Rudy et al. [114] showed a tenfold higher sodium content in koshered beef compared with meat obtained from conventional slaughter, regardless of gender and muscle type. Mast and Macneil [115], using shear tests and sensory techniques, reported that koshered chicken breast meat (with a 4–6 times higher sodium content) was more tender compared with the nonkoshered raw material, which resulted in a higher overall acceptability rating.

## 5. Conclusions

The results of this study indicate that each of the studied kosher determinants, i.e., slaughter type, gender, and muscle type, affects beef quality characteristics. For example, the koshering process darkens the beef and lowers the share of the colors red (a*) and yellow (b*). Koshered beef is characterized by decreased adhesiveness, gumminess, and chewiness. Hence, for a consumer who is not a religious Jew, koshered beef might not be an appealing product. However, in both muscles studied, the koshering process had a positive effect on tenderness, juiciness, and palatability. Furthermore, young bull meat is characterized by a stronger aroma intensity, whereas heifer meat is more tender regardless of slaughter type, and MLT was the juiciest.

## Figures and Tables

**Table 1:** Beef color parameters ( $\bar{x}$ ± SE) depending on slaughter type, muscle type, and gender of cattle (2 slaughter types × 2 gender groups × 2 muscle groups × 20 carcasses = 160 samples).

Specification	Muscle Type	Standard Slaughter		Kosher Slaughter		ANOVA
		Young Bulls	Heifers	Young Bulls	Heifers	
L*_48_	MLT	42.34 ^a^ ± 3.21	39.06 ^a^ ± 3.47	33.55 ^b^ ± 4.57	34.58 ^b^ ± 4.94	S *
	MS	41.32 ^a^ ± 2.86	40.47 ^a^ ± 3.79	36.20 ^b^ ± 2.99	35.17 ^b^ ± 4.48	
a*_48_	MLT	21.31 ^A^ ± 2.05	22.06 ^Aa^ ± 2.38	19.90 ± 5.13	18.76 ^b^ ± 4.01	M *; S *
	MS	24.46 ^Ba^ ± 1.56	24.59 ^Ba^ ± 3.04	19.28 ^b^ ± 6.68	20.32 ^b^ ± 3.19	
b*_48_	MLT	8.38 ± 1.64	8.74 ± 1.86	7.14 ± 5.04	7.57 ± 2.33	S *
	MS	9.50 ^a^ ± 1.50	10.06 ^a^ ± 2.18	6.23 ^b^ ± 5.15	7.88 ± 1.68	
L*_72_	MLT	41.44 ^a^ ± 3.83	39.11 ± 4.57	36.49 ^b^ ± 3.73	37.90 ^b^ ± 3.97	S *; G × S *
	MS	40.09 ^a^ ± 3.04	39.83 ^a^ ± 3.82	35.57 ^b^ ± 4.41	36.71 ^b^ ± 2.76	
a*_72_	MLT	26.23 ^Aa^ ± 1.99	26.81 ^Aa^ ± 2.03	20.91 ^b^ ± 2.78	22.22 ^b^ ± 1.85	M *; S *; M × S *
	MS	28.33 ^Ba^ ± 2.19	28.37 ^Ba^ ± 2.54	21.35 ^b^ ± 3.26	21.02 ^b^ ± 3.24	
b*_72_	MLT	11.67 ^a^ ± 1.14	12.10 ^a^ ± 1.08	7.59 ^b^ ± 1.65	8.75 ^c^ ± 1.43	S *; M × S *
	MS	12.18 ^a^ ± 1.00	12.79 ^a^ ± 1.30	7.25 ^b^ ± 2.46	7.26 ^b^ ± 1.71	
ΔL	MLT	−0.90	0.05	2.94	3.32	–
	MS	−1.23	−0.64	−0.63	1.54	
Δa	MLT	4.92	4.75	1.01	3.46	–
	MS	3.87	3.78	2.07	0.70	
Δb	MLT	3.29	3.36	0.45	1.18	–
	MS	2.68	2.73	1.02	−0.62	
ΔE	MLT	5.99	5.82	3.14	4.94	–
	MS	4.87	4.71	2.39	1.80	

^a,b,c^ Differences indicated in the rows with statistically significant values at *p* < 0.05 according to Tukey’s HSD test. ^A,B^ Differences indicated in the columns only between the muscles with statistically significant values at *p* < 0.05 according to Tukey’s HSD test. No letters or the same letters indicate no statistically significant differences. ANOVA: three-factor analysis of variance between slaughter type (S), gender (G), and muscle type (M) * *p* < 0.05. MLT: longissimus thoracis muscle; MS: supraspinatus muscle.

**Table 2:** Beef texture parameters ( $\bar{x}$ ± SE) depending on slaughter type, muscle type, and gender of cattle (2 slaughter types × 2 gender groups × 2 muscle groups × 20 carcasses = 160 samples).

Specification	Muscle Type	Standard Slaughter		Kosher Slaughter		ANOVA
		Young Bulls	Heifers	Young Bulls	Heifers	
Hardness 1 (N)	MLT	84.71 ^Aa^ ± 20.15	121.19 ^A^ ± 21.42	140.16 ^b^ ± 27.93	107.16 ± 28.21	G × M *; G × M × S *
	MS	93.34 ^Ba^ ± 20.89	136.77 ^B^ ± 26.50	102.58 ± 20.04	173.34 ^b^ ± 23.35	
Hardness 2 (N)	MLT	58.65 ^a^ ± 17.50	74.29 ± 13.64	93.15 ^b^ ± 20.50	73.26 ± 17.50	G × M × S *
	MS	54.72 ^a^ ± 11.78	76.59 ± 13.65	67.90 ± 10.15	106.80 ^b^ ± 18.66	
Rigidity 1 (N)	MLT	9.14 ± 1.98	16.27 ^A^ ± 3.59	14.78 ± 2.60	12.85 ± 2.63	G × M *
	MS	10.76 ± 2.60	23.77 ^Ba^ ± 3.82	5.98 ^b^ ± 0.89	20.78 ± 3.68	
Rigidity 2 (N)	MLT	40.24 ^A^ ± 7.94	71.86 ^A^ ± 14.69	66.63 ± 16.21	59.87 ± 11.43	G × M *; G × M × S *
	MS	44.43 ^Ba^ ± 8.73	78.42 ^B^ ± 15.35	30.70 ^a ^± 5.49	96.42 ^b^ ± 15.58	
Adhesiveness (mJ)	MLT	2.82 ± 0.65	4.14 ^A^ ± 0.60	3.86 ± 0.67	4.28 ^A^ ± 0.95	M *; S *; G × M *; G × S *
	MS	2.30 ^a^ ± 0.51	2.63 ^B^ ± 0.61	4.37 ^b^ ± 1.21	2.96 ^B^ ± 0.40	
Resilience	MLT	0.20 ^a^ ± 0.04	0.13 ^b^ ± 0.02	0.17 ± 0.03	0.18 ± 0.03	–
	MS	0.14 ± 0.01	0.15 ± 0.03	0.18 ± 0.03	0.17 ± 0.01	
Cohesiveness	MLT	0.31 ^a^ ± 0.06	0.21 ^b^ ± 0.02	0.26 ± 0.03	0.29 ± 0.04	–
	MS	0.24 ± 0.06	0.41 ± 0.08	0.25 ± 0.05	0.28 ± 0.05	
Springiness (mm)	MLT	3.37 ± 0.78	3.60 ± 0.33	4.43 ± 1.03	3.85 ± 0.30	–
	MS	3.25 ± 0.87	5.72 ± 1.04	2.66 ± 0.53	4.85 ± 0.69	
Gumminess (N)	MLT	24.86 ± 5.03	23.63 ^Aa^ ± 3.92	36.56 ^b^ ± 7.42	28.73 ^A^ ± 6.99	S *; G × M *
	MS	22.51 ^a^ ± 4.96	38.81 ^B^ ± 6.10	31.81 ± 6.11	49.62 ^Bb^ ± 8.49	
Chewiness (mJ)	MLT	107.14 ± 26.04	86.75 ^A^ ± 19.14	141.46 ± 33.23	113.58 ^A^ ± 30.23	S *; G × M *
	MS	102.08 ^a^ ± 22.56	127.32 ^Ba^ ± 24.29	119.13 ^a^ ± 27.29	254.91 ^Bb^ ± 31.84	

^a,b^ Differences indicated in the rows with statistically significant values at *p* < 0.05 according to Tukey’s HSD test. ^A,B^ Differences indicated in the columns only between the muscles with statistically significant values at *p* < 0.05 according to Tukey’s HSD test. No letters or the same letters indicate no statistically significant differences. ANOVA: three-factor analysis of variance between slaughter type (S), gender (G), and muscle type (M) * *p* < 0.05. MLT: longissimus thoracis muscle; MS: supraspinatus muscle.

**Table 3:** Sensory properties of beef ( $\bar{x}$ ± SE) depending on slaughter type, muscle type, and gender of cattle (2 slaughter types × 2 gender groups × 2 muscle groups × 20 carcasses = 160 samples).

Specification	Muscle Type	Standard Slaughter		Kosher Slaughter		
		Young Bulls	Heifers	Young Bulls	Heifers	ANOVA
Aroma: Intensity	MLT	3.90 ^a^ ± 0.29	3.67 ^b^ ± 0.76	3.93 ^a^ ± 0.29	3.50 ^b^ ± 0.001	G *
	MS	3.93 ^a^ ± 0.29	3.60 ^b^ ± 0.50	3.90 ^a^ ± 0.87	3.56 ^b^ ± 0.90	
Aroma: Desirability	MLT	3.83 ± 0.76	3.65 ± 0.58	3.70 ± 1.53	3.58 ± 0.001	–
	MS	3.90 ± 0.29	3.60 ± 0.76	3.83 ± 0.76	3.60 ± 0.29	
Tenderness	MLT	3.21 ^Aa^ ± 1.00	4.15 ^Ab^ ± 0.58	4.10 ^Ab^ ± 1.00	4.61 ^Ac^ ± 1.04	S *; G *; M *; S × G *; G × M *
	MS	2.80 ^Ba^ ± 0.50	3.70 ^Bb^ ± 0.29	3.63 ^Bb^ ± 1.23	4.25 ^Bc^ ± 0.29	
Juiciness	MLT	3.33 ^a^ ± 0.58	3.90 ^Ab^ ± 0.10	4.40 ^Ac^ ± 0.58	4.76 ^Ac^ ± 0.76	S *; M *; S × G *; G × M *
	MS	3.00 ^a^ ± 0.90	3.47 ^Bb^ ± 0.29	3.90 ^Bc^ ± 1.04	4.30 ^Bc^ ± 0.58	
Taste: Intensity	MLT	3.77 ^a^ ± 0.58	3.80 ^a^ ± 0.50	4.60 ^b^ ± 1.73	4.73 ^b^ ± 0.58	S *
	MS	3.66 ^a^ ± 0.29	3.70 ^a^ ± 0.00	4.34 ^b^ ± 1.00	4.67 ^b^ ± 0.29	
Taste: Desirability	MLT	3.67 ^a^± 0.58	3.70 ^a^ ± 0.50	4.47 ^b^ ± 1.00	4.53 ^b^ ± 0.58	S *
	MS	3.41 ^a^ ± 0.29	3.57 ^a^ ± 0.29	4.10 ^b^ ± 1.00	4.43 ^b^ ± 0.29	
General Acceptability	MLT	3.62 ± 0.57	3.81 ± 0.13	4.20 ± 0.89	4.29 ± 0.48	–
	MS	3.45 ± 0.14	3.61 ± 0.27	3.95 ± 0.97	4.14 ± 0.25	

^a,b,c^ Differences indicated in the rows with statistically significant values at *p* < 0.05 according to Tukey’s HSD test. ^A,B^ Differences indicated in the columns only between the muscles with statistically significant values at *p* < 0.05 according to Tukey’s HSD test. No letters or the same letters indicate no statistically significant differences. ANOVA: three-factor analysis of variance between slaughter type (S), gender (G), and muscle type (M) * *p* < 0.05. MLT: longissimus thoracis muscle; MS: supraspinatus muscle.

## Data Availability

The authors declare that data or models are not deposited in an official repository.
